# The Management of Immunosuppression in Kidney Transplant Recipients with COVID-19 Disease: An Update and Systematic Review of the Literature

**DOI:** 10.3390/medicina57050435

**Published:** 2021-04-30

**Authors:** Roberta Angelico, Francesca Blasi, Tommaso Maria Manzia, Luca Toti, Giuseppe Tisone, Roberto Cacciola

**Affiliations:** 1 Department of Surgery Sciences, Transplant and HPB Unit, University of Rome Tor Vergata, 00133 Rome, Italy; roberta.angelico@uniroma2.it (R.A.); francesca.b1991@gmail.com (F.B.); toti@med.uniroma2.it (L.T.); tisone@uniroma2.it (G.T.); rc.1968@icloud.com (R.C.); 2 Department of Surgery, King Salman Armed Forces Hospital, Tabuk 47512, Saudi Arabia

**Keywords:** coronavirus disease 2019, severe acute respiratory syndrome coronavirus 2, kidney transplantation, immunosuppression, complications, allograft outcomes, patient outcomes

## Abstract

*Background and Objectives:* In the era of the coronavirus disease 2019 (COVID-19) pandemic, the management of immunosuppressive (IS) therapy in kidney transplant (KT) recipients affected by severe acute respiratory syndrome coronavirus 2 (SARS-CoV-2) requires attention. It is not yet understood whether IS therapy may protect from the cytokine storm induced by SARS-CoV-2 infection or a temporary adjustment/withdrawal of IS therapy to restore the immune system may be necessary. We performed a systematic literature review to investigate the current management of IS therapy in KT recipients with COVID-1. *Materials and Methods:* Out of 71 articles published from 1 February 2020 until 30 October 2020, 554 KT recipients with SARS-CoV-2 infection were identified. *Results:* Modifications of IS therapy were based on the clinical conditions. For asymptomatic patients or those with mild COVID-19 symptoms, a “wait and see approach” was mostly used; a suspension of antimetabolites drugs (347/461, 75.27%) or mTOR inhibitors (38/48, 79.2%) was adopted in the majority of patients with symptomatic COVID-19 infections. For CNIs, the most frequent attitude was their maintenance (243/502, 48.4%) or dose-reduction (99/502, 19.72%) in patients asymptomatic or with mild COVID-19 symptoms, while drug withdrawal was the preferred choice in severely symptomatic patients (160/450, 31.87%). A discontinuation of all IS drugs was used only in severely symptomatic COVID-19 patients on invasive mechanical ventilation. Renal function remained stable in 422(76.17%) recipients, while 49(8.84%) patients experienced graft loss. Eight (1.44%) patients experienced a worsening of renal function. The overall mortality was 21.84%, and 53(9.56%) patients died with functioning grafts. *Conclusion:* A tailored approach to the patient has been the preferred strategy for the management of IS therapy in KT recipients, taking into account the clinical conditions of patients and the potential interactions between IS and antiviral drugs, in the attempt to balance the risks of COVID-19-related complications and those due to rejection or graft loss.

## 1. Introduction

Kidney transplantation (KT) is the best choice for patients with end-stage renal disease. During the past decades, patient and graft survival after KT has considerably improved [1,2], mainly due to the availability of new immunosuppressive (IS) drugs. However, if on one hand, IS agents are necessary to prevent rejection, on the other hand, they increase the risk of infections. These infections, indeed, remain the most common non-cardiovascular causes of death after KT [3,4]. 

The severe acute respiratory syndrome coronavirus 2 (SARS-CoV-2) was first identified in humans during December 2019 as the cause of coronavirus disease 2019 (COVID-19). The manifestations of COVID-19 are different from patient to patient, ranging from asymptomatic/mild symptoms to severe illness leading to death; the main symptoms are fever, weakness, respiratory distress, muscle pain, and loss of taste or smell [5]. Strong international recommendations have been generated to prevent the transmission of SARS-CoV-2, which include the use of personal protective devices, avoiding exposure in high-risk places, frequent hand washing, wearing of masks, and social distancing measures [6]. Despite this, the risk of developing COVID-19 in transplant patients is higher than in the general population. Recently, the US Centers for Disease Control and Prevention has classified KT recipients as a high-risk group for severe COVID-19 [7], due to both the immunocompromised status and impaired kidney function. 

A few articles have focused so far on the management of IS therapy in KT recipients with COVID-19 [8]. Generally, a reduction in IS drugs is a widely used strategy for the management of other viral infections, which may occur in both kidney and liver transplant recipients. The 2009 KDIGO guidelines for the management of IS therapy after KT recommend to use a combination of IS drugs, including CNI and ADs (with a level 1 recommendation) as standard therapy, and suggest their adjustment in case of viral infection [9]. Despite these recommendations mention the therapeutic strategies to be adopted in case of viral infections from BK virus or cytomegalovirus, Epstein–Barr virus, Herpes virus, hepatitis viruses, or human immunodeficiency virus, yet there are currently no evidence-based guidelines for managing IS regimens in patients testing positive for SARS-CoV-2; there are only expert opinions [8,9]. The present study aims to review the management of IS drugs in KT recipients infected by SARS-CoV-2 in order to provide practical tools for the administration of IS therapy in KT recipients with SARS-CoV-2 infection, taken from the best of the available literature. 

## 2. Materials and Methods

### 2.1. Search Strategy

A systematic research was performed to identify relevant studies focused on the management of IS drugs in adult KT recipients infected with SARS-CoV-2. The search strategy complied with the Preferred Reporting Items for Systematic Reviews and Meta-Analyses (PRISMA) guidelines [10].

A systematic search within the MEDLINE, PubMed, and Embase electronic databases was carried out to identify relevant English-language articles published from 1 February 2020 to 30 October 2020. Search terms included coronavirus, COVID-19, SARS-CoV-2, renal transplantation, and kidney transplantation. 

### 2.2. Screening Process

The present systematic review included an a priori search criteria of journal articles amongst adult (≥18 years old) KT recipients infected with SARS-CoV-2. In the search, we included all original articles, case reports, case series, case-control studies, and correspondence articles dealing with KT recipients infected by SARS-CoV-2 that detailed the management of IS drugs. Studies lacking the details of IS therapy were excluded. All studies that originated from the same center were considered and analyzed, and possible overlap of clinical cases reported across the studies were evaluated.

### 2.3. Study Selection and Data Extraction

**Figure 1:**
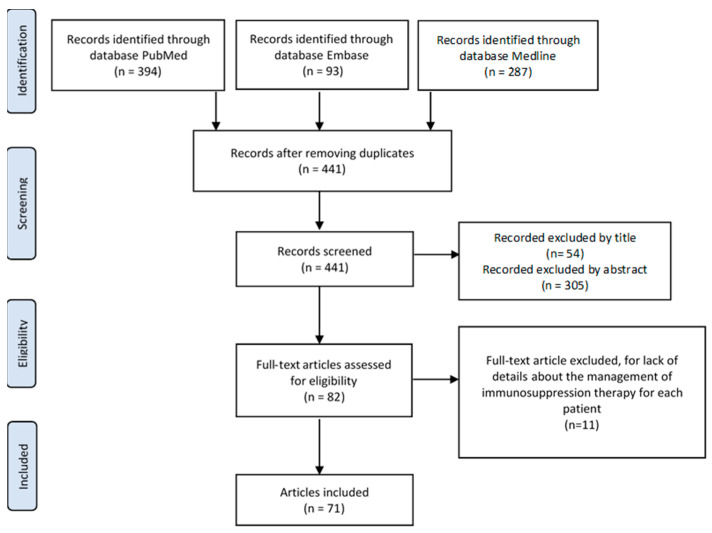
Flowchart of the literature search and study selection.

A total of 821 articles were identified. Two reviewers (R.A. and F.B.) independently screened the identified studies and their data were extracted. In case of disagreement, the paper was discussed by all the authors. After the systematic screening, 71 articles were identified for the systematic review analysis (Figure 1), including 48 case reports, 21 case series, 1 case-control study, and 1 prospective study as summarized in Table 1 (reporting case reports with a maximum of two patients) and Table 2 (describing articles with more than two patients). Given the paucity of patients identified within the selection criteria in each selected article, the results are reported as a narrative review. Data extracted from each study [11,12,13,14,15,16,17,18,19,20,21,22,23,24,25,26,27,28,29,30,31,32,33,34,35,36,37,38,39,40,41,42,43,44,45,46,47,48,49,50,51,52,53,54,55,56,57,58,59,60,61,62,63,64,65,66,67,68,69,70,71,72,73,74,75,76,77,78,79,80,81] included year of publication, number of participants, and the data-relevant outcome variables explained above.

### 2.4. Study Quality Assessment

Included manuscripts were evaluated for methodological quality using the modification of the Newcastle–Ottawa Quality Assessment Scale and the tool provided by Murad and colleagues, as appropriate [82]. Only questions relevant to the review purpose were considered. We opted for an overall judgement and the quality of the selected studies was classified as low, average, or high (Supplementary Table S1). 

### 2.5. Statistical Analysis

Due to the nature and quality of the studies available, no meta-analysis could be performed. In order to offer a compact description of the literature, categorical variables were reported as numbers, whereas continuous variables were reported as ranges. Therefore, the summary provided must be considered as a narrative description, and no inferences should be drawn.

## 3. Results

Our analysis identified 554 KT recipients with confirmed SARS-CoV-2 infection, for whom the management of IS therapy was reported (Table 1 and Table 2). The median age of KT recipients at time of detection of SARS-CoV-2 positivity was 46 (range: 31–75) years, and the median time from transplantation was 6.1 (range: 0–25) years. 

At the time of detection of SARS-CoV-2 positivity, the maintenance IS therapy was mostly based on calcineurin inhibitors (CNIs) (n = 502, 90.61%), including tacrolimus (Tac) (n = 453, 81.76%), cyclosporine A (CyA) (n = 28, 5.05%), or non-specified CNI (n = 21, 3.79%)], followed by antimetabolites drugs (ADs) (n = 461, 83.2%) and mammalian target of rapamycin (mTOR) inhibitors (n = 48, 8.66%). At the time of SARS-CoV-2 detection, 399 (72.02%) patients were receiving steroids.

The overall management of IS therapy in KT recipients affected by COVID-19 is shown in Table 3. The most frequent approach consisted of the discontinuation of ADs (347/461, 75.27%), while ADs were maintained stable in 70/461 (15.18%) patients or reduced in 44/461 (9.54%) cases. Administration of CNIs was unchanged in 243/502 (48.4%) KT recipients, while it was reduced in 99/502 (19.72%) KT recipients and discontinued in 160/502 (31.87%) KT recipients. Among 453 KT recipients receiving Tac, in 229 (50.6%) KT recipients Tac was not modified, in 138 (30.5%) patients, it was suspended, and in 86 (19.0%), it was reduced. Thus, CyA was suspended in more than half of the patients (17/28, 60.7%) and not modified in the remanent patients (11/28, 39.3%). Regarding mTOR inhibitors, which were less frequently used, these were discontinued in 79.2% (38/48) of infected patients. 

Four (0.8%) KT recipients developed a SARS-CoV-2 infection within 6 months from KT [30,44]. In all of them, the induction therapy had consisted of thymoglobulin, and all patients were receiving steroids at the time of the detection of the SARS-CoV-2 infection. Of these, two patients were managed with the withdrawal of ADs and two cases were managed with the reduction in ADs. Tac was reduced in all of them, except for one patient in whom it was completely discontinued. All patients were alive, and none developed deterioration of renal function that required hemodialysis. 

For a large majority of asymptomatic KT recipients or those with mild symptoms, no modifications of the IS regimen were made. In contrast, in KT recipients with symptomatic COVID-19, suspension of ADs or mTOR inhibitors was adopted in up to 79% of patients, especially in those patients requiring invasive ventilation, while CNI withdrawal (31.87%) was reserved in those who were severely symptomatic. A complete discontinuation of all IS drugs was adopted only in KT recipients with COVID-19 receiving invasive mechanical ventilation. 

All SARS-CoV-2-infected KT recipients underwent strict monitoring of their clinical conditions and renal function. Kidney graft function remained stable in 422 (76.17%) patients, 49 (8.84%) patients experienced graft loss and 8 (1.44%) patients developed worsening renal function caused by acute kidney injury. In 67 (12.09%) KT recipients, the graft outcome was not reported. 

Overall, 121 (21.84%) KT recipients with COVID-19 died. Of these, 53 (43.8%) patients died with a functioning graft, 41 (33.8%) patients died with a non-functioning graft, and two (1.65%) patients died with impaired renal function not requiring dialysis, while in 21 (17.35%) cases, the graft status at the time of death was not detailed. 

Considering all the cases described in the literature, it was found that 49 (8.84%) KT recipients with COVID-19 required replacement renal treatment and 45 (91.83%) of these patients died.

## 4. Discussion

Since the start of the COVID-19 pandemic, as of 21 April 2021, there have been 142 million cases and 3.032 million deaths have been reported. So far, studies investigating the impact of the pandemic on the use of IS therapy in KT recipients and their related outcomes are limited. Elias et al. [67] reported the French experience, which represents the largest cohort of KT recipients affected by COVID-19 (n = 66) so far described. In this prospective study, 29 (43.9%) patients underwent a reduction in their IS regimen, which was more frequently done in patients requiring invasive mechanical ventilation (87%) than in those not requiring respiratory support (57%). As in other series, the primary modification of IS therapy consisted of withdrawal of ADs (62%) while continuing CNIs and the baseline steroids (for those individuals who were on maintenance prednisolone). In the subgroup of patients requiring invasive mechanical ventilation (n = 15), ADs were suspended in all patients. Thus, CNIs were discontinued in only 14% of them. This IS management adopted in the French experience reflects the common approach found in the majority of the articles analyzed. The overall mortality rate of the French report was 24% (16/66), which is in line with the results of the present study. However, the mortality was definitely higher in patients requiring invasive mechanical ventilation (11/15, 73%) compared to that observed in patients without invasive respiratory support (5/51, 10%).

Another large experience was described by Pierotti et al. [77], who reported a retrospective study of 51 KT recipients with COVID-19 in Sao Paulo, Brazil. In the majority of patients (32/51, 62.7%), ADs and mTOR inhibitors were withdrawn, while CNIs and steroids were maintained at reduced doses; thus, in 19/51 (37.2%) patients, mainly admitted to the ICU (17/19, 89.5%), ADs and CNIs were completely suspended, while steroids (hydrocortisone) were maintained.

Based on the current data, the risk of developing COVID-19 in transplant patients is reported to be about 5%, being higher than in the general population (0.3%) [67,83]. Some studies have suggested that the risk factors associated with COVID-19 disease in KT recipients are non-white ethnicity, obesity, asthma, chronic pulmonary disease, and diabetes, as in the general population, and in addition, the immunocompromised status and pre-existent kidney disease [67,84]. From the current available data, the severity of the systemic disease due to COVID-19 negatively affects patient survival, and in patients with very severe disease, it is even greater in both transplanted and non-transplanted patients. The mortality rate among KT recipients with COVID-19 has been reported to be between 23% and 28%, which is certainly higher than the figure of <5% mostly found in the non-transplant population of COVID-19 infected patients [53]. Accordingly, recent data from the European Renal Association-European Dialysis and Transplant Association (ERA-EDTA) Registry confirmed the vulnerability of KT recipients infected with COVID-19, who showed a mortality rate of 20–25% in the European countries more severely affected by the pandemic [85]. 

Compared to non-COVID-19 pneumonia, the figures of KT with COVID-19 are still different. In a retrospective study focusing on severe pneumonia (due to bacterial, viral, or fungal infection) requiring intensive care unit admission in 60 KT recipients, the mortality rate was 18.8% in patients with pneumonia early after transplantation and 7.1% in these with late onset of pneumonia [86]. In this study, graft function was maintained stable in the majority of patients (76.7%), while 6.7% required renal replacement therapy during the intensive care unit stay and 3.3% of these experienced graft losses [86]. These data suggest that the outcomes of non-COVID-19 pneumonia after KT seem to be slightly superior compared to the outcomes of patients affected by COVID-19.

As extensively described by Gagliardi et al. [87], the SARS-CoV-2 infection itself has been associated to the development of acute kidney injury especially in patients with a pre-existing chronic kidney damage. The two main pathophysiological mechanisms of kidney damage in COVID-19 patients are direct cytopathic effect of SARS-CoV-2 on renal epithelial cells and an indirect cytokine storm syndrome. Moreover, hypoxia, persistent hypotension, rhabdomyolysis, over activation of the coagulation cascade, and impairment of microcirculation play a role in the predisposition to the development of acute renal damage. Based on this, it is reasonable that these mechanisms may occur also in kidney grafts, worsening the renal function during the COVID-19 disease. 

Since the immunocompromised status of KT recipients may conceivably influence the outcome of COVID-19 disease in KT recipients, the management of the IS regimen is thought to play a central role in such frail patients. Some data suggest that IS may exert “a protective role”, as the cytokine storm is considered to be an important factor in the pathogenesis of the disease. Yet, on the other hand, it is also reasonable to advise a reduction, or a temporary withdrawal, of IS drugs during a severe course of COVID-19 in order to obtain earlier restoration of the host immune system [88,89]. 

The concomitance of increased COVID-19-related morbidity with the risks of acute rejection and graft loss makes the management of IS therapy in KT recipients infected with SARS-CoV-2 a very difficult task, which does not currently rely on solid evidence. In this review, we therefore aimed to assess how transplant physicians are currently handling the use of IS drugs in KT recipients with COVID-19 to the best of the available literature. We found that maintenance IS therapy, at the time of detection of SARS-CoV-2 positivity, was mainly based on CNIs, ADs, and mTOR inhibitors, with or without steroids. Most importantly, all the authors of the articles that we identified advised to consider an individual modification of IS therapy based on the clinical condition of each patient. 

For KT recipients with mild symptoms, the “wait and see approach” was the most commonly used [44,90]. This conservative clinical practice is in line with the recommendation of the Developing Education Science and Care for Renal Transplantation in European States (DESCARTES) Working Group, an expert panel of the ERA-EDTA [8]. This is also similar to the recommendations of the American Association for the Study of Liver Diseases (AASLD) for SAR-COV-2-infected KT recipients, supporting the view that there is no need to discontinue, nor to decrease, the dose of immunosuppressant in non-critically ill patients [91]. 

In contrast, in KT recipients with symptomatic COVID-19, the most frequently used approach was the suspension of the ADs or mTOR inhibitors, regardless the fact that both these IS drugs showed in vitro antiviral effects against some type of coronavirus. In addition, there is evidence that they inhibit T cell function and proliferation [92] and interfere in the regulation of the proteins involved in the cell cycle, angiogenesis, and glycolysis [93]. 

The ADs include mycophenolate mofetil (MMF), mycophenolic acid (MPA), and azathioprine (AZA). MMF, a derivative of mycophenolic acid, is a selective inhibitor of purine synthesis, determining a potent inhibition of T and B cell proliferation. MPA showed, in in vitro studies, an antiviral effect on four subtypes of coronaviruses, but not for COVID-19 [94]. However, MMF has well-known adverse hematologic effects, including leukopenia and thrombocytopenia, which may exacerbate hematologic complications of COVID-19 [95]. AZA is an imidazole derivative antimetabolite of 6-mercaptopurine. It inhibits DNA synthesis, thus blocking the proliferation and differentiation of T and B lymphocytes and the production of antibodies and decreasing the level of circulating monocytes and granulocytes. Therefore, these actions of the ADs might compromise the immune response against COVID-19.

Differently, the mTOR inhibitors, namely, everolimus and sirolimus, bind to the intracellular protein FKBP-12, forming a complex that inhibits the activity of mTOR complex-1 (mTORC1) and mTORC2. This inhibition interferes with translation and protein synthesis by reducing the activity of ribosomal protein kinase S6 (S6K1) and eukaryotic translation elongation factor 4E binding protein (4EBP-1), which regulate the proteins involved in the cell cycle, angiogenesis, and glycolysis. In vitro mTOR inhibitors have been shown to reduce the replication of another coronavirus, the Middle East respiratory syndrome-related coronavirus (MERS-CoV) [96]. However, at the same time, mTOR inhibitors can induce leukopenia, thrombocytopenia, and pneumonitis, therefore possibly further worsening the hematological effects of COVID-19.

For the two CNIs, CyA and Tac, the most frequent approach was maintenance of the therapy or reduction in their dosage in asymptomatic or mildly symptomatic COVID-19 patients. Only in KT recipients with severe COVID-19 symptoms and in patients requiring invasive respiratory support, CNIs were completely suspended. However, there is a lack of data in the literature regarding the safe trough levels of CNIs to be adopted in KT recipients with COVID-19. 

Despite CyA and Tac having different molecular structures and intracellular characteristics, they share the same IS properties consisting of the inhibition of calcium- and calmodulin-dependent phosphatase proteins or calcineurin. CyA binds to cyclophilin, while Tac binds to FKBP12 [97,98]. Their interaction with calcineurin inhibits the phosphatase activity of calcineurin, and in this way, they suppress transcription of IL-2 by impairing the translocation of the nuclear factor of activated T cells (NFAT), which leads to a non-response of T lymphocytes to specific antigenic stimuli [99]. Some studies showed that both CyA and Tac might exert antiviral activity, as evidenced in vitro on different coronavirus strains [92,100]. Interestingly, in a recent Italian study [93], patients treated with CNIs affected by SARS-CoV-2 showed a low severity of COVID-19 infection. The authors speculated that this apparently more favorable clinical course might be related to the long-term exposure of patients to the IS regimens, which might have prevented the occurrence of massive alveolar macrophage activation with lower release of pro-inflammatory cytokines, which play a key role in the context of COVID-19 infection [101]. Taken together, these data suggest that CNIs may indeed exert a certain degree of antiviral activity against SAR-CoV-2, an interesting hypothesis that obviously needs confirmation. Despite both Tac and mTOR inhibitors binding FKBP12, there are no sufficient data to explain why the majority of the authors preferred to suspend the mTOR inhibitors more frequently than Tac; one explanation could be related to the fact that Tac is the most frequently used IS drug in KT recipients, its management is better known, and it is also the most active in preventing allograft rejection. Moreover, m-TOR inhibitors can induce leukopenia and thrombocytopenia, which can cause a worsening of the COVID-19 hematological effects.

On the other hand, possible interactions between CNIs or mTOR inhibitors and antiviral drugs commonly used against COVID-19, such as lopinavir or ritonavir, have been well demonstrated; hence, a strict monitoring of these IS drugs is mandatory [102]. The doses of CNIs and mTOR inhibitors should be reduced or the two drugs should be withdrawn according to the duration of the antiviral treatment, as also suggested by the DESCARTES Working Group [8].

Lastly, also the use of corticosteroids for the management of COVID-19 is controversial. The Randomized Evaluation of COVID-19 Therapy (RECOVERY) trials showed benefits of high doses of dexamethasone in patients under mechanical ventilation [103]; meanwhile, a recent systematic review showed a longer period of hospitalization for patients who received glucocorticoids, without benefits in terms of mortality [103]. It is well-known that corticosteroids are involved in a number of key physiological processes, including the immune response and inflammation, modulating a variety of cytokines; accordingly, their use appeared to be favorable in several viral infections [104,105]. In patients affected by SARS caused by SARS-CoV-2 infection, steroids (usually methylprednisolone) are currently administered; however, some studies have identified that steroid therapy may even prolong the viral shedding [106]. In our analysis, the majority of KT recipients, under maintenance steroid therapy, were maintained on their habitual steroid dose during the COVID-19 infection.

Despite a few data being identified on induction agents in KT recipients with COVID-19 infection [107], the induction therapy is a very relevant aspect in KT, as it is widely used to provide fast and effective protection against acute allograft rejection [108]. Yet, at the same time, induction therapy may increase the risk of infectious diseases [109,110]. The commonly used induction agents, such as anti-thymocyte globulin (ATG) and rituximab, produce powerful lymphocyte depletion, which is highly effective in preventing acute rejection [111]; however, the effects of these agents extend well beyond the first month after KT [112]. Consequently, the use of lymphocyte-depleting induction IS therapy may expose KT recipients to an increased vulnerability to COVID-19 infection. In the context of the current pandemic, non-lymphocyte-depleting agents, such as basiliximab, may be considered a more prudent choice, although its advantages have been questioned [113]. 

For new KT recipients, considering the overall IS dose (induction plus maintenance therapy) administered at the time of KT, with the available evidence of nosocomial and opportunistic infections after KT [114,115], we may counterintuitively consider that the newly immunosuppressed patients are more vulnerable to COVID-19 as compared to those on long-term maintenance regimes, but, further data are needed to confirm this assumption. In this sub-category, restricting the use of lymphocyte-depleting agents to specific circumstances and favoring the use of basiliximab may constitute a measured initial approach. Today, more than ever, in the context of a pandemic scenario, we may argue whether the use of an induction therapy in patients with a low immunological risk is really necessary [116], given that the use of a common triple IS regimen, such as the combination of Tac, MMF, and steroids, significantly reduces the risk of rejection [117,118]. On the other hand, the use of induction therapy could reduce the chronic exposure to CNIs and steroids; therefore, it is questionable if it is better to use induction therapy and low-dose maintenance therapy or not administer induction therapy followed by initial high-dose maintenance therapy [119].

Based on the current literature review and our experience, we suggest that the modification of IS regimens in KT recipients affected by COVID-19 infection needs to be evaluated case by case, according to the clinical status of the patient with strict monitoring. We believe that IS therapy should be cautiously modified only in symptomatic patients, since the current data show no benefits of a radical modification of IS therapy in patients with mild symptoms. In patients with moderate symptoms who require modification of IS therapy, we suggest to start with a reduction or discontinuation of the ADs. Differently, patients with severe symptoms, especially those with invasive respiratory support, require complete withdrawal of IS drugs, and the maintenance of steroids seems to be the best choice.

However, this debate clearly requires future exploration within a large study enrolling newly performed KT during the SARS-CoV-2 pandemic. In fact, the current analysis is limited by the paucity of the current experience; moreover, the available data are not homogeneous and largely incomplete. Therefore, their interpretation should be cautious.

## 5. Conclusions

The current knowledge of the management of IS therapy in KT recipients with SARS-CoV-2 positivity is based on small series or case reports. A tailored approach to individual patients is the currently preferred choice, by evaluating the clinical status of the patients and balancing the risk of COVID-19-related complications and the risk of rejection or graft loss. We found that the most frequently used approach for the management of IS therapy is, nowadays, the discontinuation of ADs (MMF, MPA, or azathioprine) and mTOR inhibitors in all patients. In contrast, CNIs are maintained stable, or dose-reduced, in asymptomatic or mildly symptomatic patients, while complete withdrawal of CNIs is considered in symptomatic patients. A total discontinuation of all IS drugs is commonly used only in patients with severe symptomatic COVID-19 infection requiring invasive mechanical ventilation. The careful use of corticosteroids should be adopted. Antiviral drugs commonly used for the treatment of COVID-19 may have several drug–drug interactions, causing increased serum levels of CNIs and mTOR inhibitors. These may result in adverse reactions, which may require the suspension of IS therapy. Therefore, currently available data suggest that, in KT recipients with COVID-19, any modification of IS therapy should be individualized and careful monitoring of IS drugs serum levels is advisable. However, because of the paucity of today’s experiences, further data are needed before conclusive clinical recommendations for the administration of IS therapy in new KT recipients affected by COVID-19 can be done.

## Figures and Tables

**Table 1:** Case reports of kidney transplant recipients infected with severe acute respiratory syndrome coronavirus 2 (SARS-CoV-2) included in the analysis.

Authors	Region/Country	N° pts	Type of Study	IS Modifications	Outcomes	HD Necessity	Graft Loss
[11] **Akdur et al.**	Ankara, Turkey	1	Case Report	Withdrawal (AD+Tac)	Alive	No	No
[12] **Allam et al.**	Fort Worth, TX, USA	1	Case Report	AD withdrawal, Tac reduction	Alive	No	No
[13] **Bartiromo et al.**	Florence, Italy	1	Case Report	Tac withdrawal	Alive	No	No
[14] **Billah et al.**	New York, NT, USA	1	Case Report	Tac reduction (AD stable)	Alive	Yes	Yes
[15] **Bussalino et al.**	Genova, Italy	1	Case Report	None (Tac+AD stable)	Alive	No	No
[16] **Chen et al.**	Wuhan, China	1	Case Report	Withdrawal (Tac+AD)	Alive	No	No
[17] **Cheng et al.**	Nanjiing, China	2	Case Report	Withdrawal (Tac+AD)	Alive	No	No
[18] **Chenna et al.**	Albany, NY, USA	1	Case Report	Withdrawal (Tac+AD)	Died	No	DwGF
[19] **Dahl et al**	Aarhus N, Denmark	1	Case Report	None (Tac stable)	Alive	No	No
[20] **Dirim et al.**	Istanbul, Turkey	1	Case Report	AD withdrawal, Tac reduction	Died	Yes	Yes
[21] **Fontana et al.**	Modena, Italy	1	Case Report	CyA withdrawal	Alive	No	No
[22] **Gandolfini et al.**	Parma, Italy	2	Case Report	Withdrawal (Tac+AD)	Alive (1)/ Died (1)	No	DwGF (1) No (1)
[23] Guillen et al.	Barcelona, Spain	1	Case Report	Withdrawal (Tac+mTOR)	Alive	No	No
[24] **Hasan Ahmad et al.**	Ipswich, UK	1	Case Report	Withdrawal (AD+BELAT)	Alive	No	No
[25] **Hsu et al.**	Los Angeles, CA, USA	1	Case Report	AD withdrawal, Tac stable	Alive	No	No
[26] **Huang et al.**	Fuzhou, China	1	Case Report	Withdrawal (AD)	Died	Yes	Yes
[27] **Jiang et al.**	Wuhan, China	1	Case Report	CyA Withdrawal, AD reduction	Alive	NA	NA
[28] **Kates et al.**	Seattle, WA, USA	1	Case Report	AD withdrawal, Tac reduction	Alive	No	No
[29] **Kemmner et al.**	Munich, Germany	1	Case Report	AD withdrawal, CyA introduction	Alive	No	No
[30] **Kim et al.**	Daegu, Korea	2	Case Report	Tac withdrawal (1)/Tac stable (1), AD withdrawal (2)	Alive	No	No
[31] **Kocak et al.**	Istanbul, Turkey	2	Case Report	AD withdrawal (2), Tac reduced (1)/ Tac withdrawal (1)	Alive	No	No
[32] **Kolonko et al.**	Katowice, Poland	3	Case Report	AD withdrawal (1)/AD reduction (2), Tac stable (2)/Tac reduction (1)	Died (1)/ Alive (2)	No	No (2) DwGF (1)
[33] **Kumar et al.**	Chicago, IL, USA	1	Case Report	Reduction (Tac+AD)	Alive	No	No
[34] **Lauterio et al.**	Milan, Italy	1	Case Report	Withdrawal (CyA+mTORi)	Alive	No	No
[35] **Li.Q**	Peking, China	2	Case Report	CyA withdrawal (1) Tac withdrawal (1), AD withdrawal (1)	Died (1)	No	Worsening (1)
[36] **Ma et al.**	Hong Kong	1	Case Report	Tac withdrawal, AD reduction	Alive	No	No
[37] **Machado et al.**	Sao Paulo, Brazil	1	Case Report	Withdrawal (Tac+AD)	Alive	No	No
[38] **Man et al.**	Wuhan, China	1	Case Report	Withdrawal (Tac+AD)	Alive	No	No
[39] **Marx et al.**	Strasbourg, France	1	Case Report	Withdrawal (AD+BELAC)	Alive	No	No
[40] **Meziyerh et al.**	Leiden, the Netherlands	1	Case Report	Withdrawal (mTORi)	Alive	No	Worsening
[41] **Namazee et al.**	Semnan, Iran	1	Case Report	Withdrawal (CyA+AD)	Died	No	Yes
[42] **Ning et al.**	Hefei, China	1	Case Report	Stable (CyA+AD)	Alive	No	No
[43] **Sakulkonkij et al.**	Lampang, Thailand	1	Case Report	Tac reduction, AD withdrawal	Alive	No	No
[44] **Seminari et al.**	Pavia, Italy	1	Case Report	Stable (Tac+AD)	Alive	No	No
[45] **Shingare et al.**	Mumbai, India	2	Case Report	Reduction (Tac+AD)	Alive	No	No
[46] **Sj Antony et al.**	El Paso, TX, USA	1	Case Report	Withdrawal (Tac+AD)	Alive	No	No
[47] **Suwanwongse et al.**	New York, NY, USA	1	Case Report	Tac withdrawal, AD stable	Died	Yes	Yes
[48] **Tanaka et al**	Osaka, Japan	1	Case Report	Tac stable, AD withdrawal, mTORi withdrawal	Alive	No	No
[49] **Tantisattamo et al.**	Orange, CA, USA	1	Case Report	AD withdrawal, Tac stable	Alive	No	No
[50] **Thammathiwat et al.**	Bangkok, Thailand	1	Case Report	Withdrawal (Tac+AD)	Alive	No	No
[51] **Tzukert T. et al.**	Jerusalem, Israel	2	Case Report	Tac stable (2), AD withdrawal (1)/AD stable (1)	Alive	No	No
[52] **Velioglu et al.**	Istanbul, Turkey	1	Case Report	AD withdrawal, Tac stable	Alive	No	No
[53] **Wang et al.**	Zhengzhou, China	1	Case Report	Stable (CyA+AD)	Alive	No	No
[54] **Wang et al.**	Stanford, CA, USA	2	Case Report	Tac stable (2), AD withdrawal (2)	Alive	No	No
[55] **Xu et al.**	Ottawa, Canada	1	Case Report	Tac withdrawal, AD stable	Alive	No	No
[56] **Zhong et al.**	Wuhan, China	1	Case Report	Reduction (Tac+AD)	Alive	No	No
[57] **Zhu et al.**	Wuhan, China	1	Case Report	Tac reduction, AD withdrawal	Alive	No	No
[58] **Zhu et al.**	Wuhan, China	1	Case Report	Tac withdrawal, AD withdrawal	Alive	No	No

**Abbreviations:** AD = antimetabolite drug, BELAT = belatacept, CyA = cyclosporine A, DwGF = death with graft functioning, IS = immunosuppressive, mTORi = mammalian target of rapamycin inhibitors, n = number, NA = not available, pts = patients, SARS-CoV-2 = severe acute respiratory syndrome coronavirus 2, Tac = tacrolimus.

**Table 2:** Case series and prospective studies of kidney transplant recipients infected with SARS-CoV-2 included in the analysis.

Authors	Region/Country	N° pts	Type of Study	IS Modifications	Mortality Rate (%)	HD Necessity (%)	Graft Loss (%)	DwFG (%)
[59] **Akalin et al.**	Bronx, NY, USA	36	Case series	AD withdrawal (24)/stable (7), Tac withdrawal (6)/stable (29)	27.7%	NA	NA	NA
[60] **Alberici et al.**	Brescia, Italy	20	Case series	IS Withdrawal [Tac (19), AD (14), mTOR (2)]	25%	5%	25%	20%
[61] **Banerjee et al.**	London, UK.	7	Case series	AD withdrawal (5)/stable (2), Tac stable (4)/reduction (1)/withdrawal (1)	14.3%	42.9%	42.9%	–
[62] **Bosch et al.**	Munich, Germany	3	Case series	AD withdrawal (3), Tac withdrawal (1), Cya started (2)	33.3%	33.3%	33.3%	–
[63] **Chen et al.**	Brooklyn, NY, USA	30	Case series	AD Withdrawal (12), Tac withdrawal (26), CyA withdrawal (3)	20%	13.3%	20%	6.7%
[64] **Columbia University**	New York, NY, USA	15	Case series	AD withdrawal (12), Tac stable (11)/reduction (2)/withdrawal (1)	6.7%	13.3%	13.3%	–
[65] **Crespo et al.**	Barcelona, Spain	16	Case series	AD withdrawal (8), mTORi withdrawal (4)/stable (1), Tac withdrawal (8)/stable (6)	50%	18.8%	18.8%	–
[66] **Devresse et al.**	Brussels, Belgium	22	Case series	AD withdrawal (18)/stable (1), Tac reduction (9)/stable (1), CyA withdrawal (2)/stable (3), mTORi withdrawal (1)/reduction (2)	9.1%	–	9.1%	9.1%
[67] Elias et al.	Paris, France	66	Prospective study	AD withdrawal (39)/stable (22), Tac withdrawal (3)/stable (54), BELAT postponed (1)/regular (5)	24.2%	10.6%	24.2%	13.6%
[68] **Fernandez-Riuz et al.**	Madrid, Spain	8	Case series	AD withdrawal (5)/reduction (1), Tac reduction (6)/stable (1), mTORi withdrawal (1)	25%	NA	NA	NA
[69] **Fung et al.**	San Francisco, CA, USA	7	Case series	AD reduction (1), Tac reduction (2)	0%	14.3%	–	–
[70] **Hartzell et al.**	New York, NY, USA	18	Case series	AD withdrawal (5)/reduction (13), Tac withdrawal (1)/stable (17)	38.9%	–	38.9%	38.9%
[71] **Lubetzky et al.**	New York, NY, USA	54	Case series	AD withdrawal (24)/reduction (15), Tac reduction (17)/stable (35)	12.96%	7.4%	5.55%	NA
[72] **Maritati et al.**	Ancona, Italy	5	Case series	AD withdrawal (4), Tac withdrawal (5), mTORi withdrawal (1)	40%	20%	40%	20%
[73] **Mehta et al.**	New York, NY, USA	34	Case series	AD withdrawal (26)/reduction (6)/stable (1), Tac stable (29), Cya stable (1), mTOR stable (1)	17.6%	–	17.6%	17.6%
[74] **Mella et al.**	Turin, Italy	6	Case series	AD withdrawal (3), Tac withdrawal (6)	66.7%	33.3%	66.7%	33.3%
[75] **Monfaret et al.**	Rasht Iran	22	Case Series	CNI withdrawal (5)/reduction (13)/stable (3), AD withdrawal (21)/stable (1), mTOR withdrawal (1)	27.3%	NA	NA	NA
[76] **Nair et al.**	New York, USA	10	Case Series	Tac stable (7)/withdrawal (2), AD stable (1)/withdrawal (8), mTORi stable (1)/withdrawal (19)	30%	10%	30%	20%
[77] **Pierotti et al.**	Sao Paulo, Brazil	51	Case series	Tac reduction (32)/withdrawal (12), AD withdrawal (32)/stable (14), mTORi withdrawal (4), CyA withdrawal (7)	25.5%	25.5%	25.5%	–
[78] **Rodriguez-Cubillo et al.**	Madrid, Spain	29	Case series	AD withdrawal (22), Tac reduction (1)/withdrawal (15), CyA start (23)/stable (6)	20.7%	10.3%	20.7%	10.3%
[79] **Silva et al.**	Porto, Portugal	5	Case series	AD withdrawal (5), Tac reduction (3)/stable (1), Cya withdrawal (1)	20%	–	20%	20%
[80] **Trujillo et al.**	Madrid, Spain	26	Case series	AD withdrawal (13)/stable (1), Tac withdrawal (4)/stable (20), mTORi withdrawal (2)/stable (5)	23.1%	–	23.1%	23.1%
[81] **Zhu et al.**	Wuhan, China	10	Case–control study	AD withdrawal (9)/stable (1), Tac withdrawal (7)/reduction (1)/stable (2)	10%	–	–	Worsening of graft function (10%)

**Abbreviations:** AD = antimetabolite drug, BELAT = belatacept, CNI = calcineurin inhibitors (used when the difference between Tac and Cya was not specified), CyA = cyclosporine A, DwGF = death with graft functioning, IS = immunosuppressive, mTORi = mammalian target of rapamycin inhibitors, n = number, NA = not available, pts = patients, SARS-CoV-2 = severe acute respiratory syndrome coronavirus 2, Tac = tacrolimus.

**Table 3:** Management of immunosuppressive drugs in kidney transplant recipients with coronavirus disease 2019 (COVID-19).

Type of IS Drugs	Total	Withdrawal	Reduction	No Modification
**CNI:**	502	160 (31.9%)	99 (19.7%)	243 (48.4%)
**-Tac**	453	138 (30.5%)	86 (19.0%)	229 (50.6%)
**-Cya**	28	17 (60.7%)	0 (0%)	11 (39.3%)
**-not specified**	21	5 (23.8%)	13 (61.9%)	3 (14.3%)
**ADs**	461	347 (75.3%)	44 (9.5%)	70 (15.2%)
**mTOR inhibitors**	48	38 (79.2%)	2 (4.1%)	8 (16.6%)

Note: Summaries based on individual cases should not be considered as an estimate of the real world. **Abbreviations**: AD = antimetabolite drugs, TAC = tacrolimus, CyA = cyclosporine A, mTOR inhibitors = mammalian target of rapamycin inhibitors.

## Data Availability

The data presented in this study are available on request from the corresponding author.

## Supplementary Materials

The following are available online at https://www.mdpi.com/article/10.3390/medicina57050435/s1, Table S1. Quality of evidence in the selected papers for the systematic review according to the Newcastle–Ottawa Scale.
