# Supplementary material for: The Management of Immunosuppression in Kidney Transplant Recipients with COVID-19 Disease: An Update and Systematic Review of the Literature
Source: Medicina (Kaunas). 2021 Apr 30;57(5):435. doi: 10.3390/medicina57050435 (PMC8147172; doi:10.3390/medicina57050435)
Supplement: Supplementary file 1 [file medicina-57-00435-s001.zip › medicina-1172311-supplementary.pdf]

Table S1. Quality of evidence in the selected papers for the systematic review according to the Newcastle-Ottawa Scale.

Author	References	Author selection (Max 4 stars)	Comparability (Max 2 stars)	Outcome/Exposure (Max 3 stars)	Total of stars
**Akdur et al.**	11	Case Report			0
**Allam et al.**	12	Case Report			0
**Bartiromo et al.**	13	Case Report			0
**Billah et al.**	14	Case Report			0
**Bussalino et al.**	15	Case Report			0
**Chen et al.**	16	Case Report			0
**Cheng et al.**	17	Case Report			0
**Chenna et al.**	18	Case Report			0
**Dahl et al**	19	Case Report			0
**Dirim et al.**	20	Case Report			0
**Fontana et al.**	21	Case Report			0
**Gandolfini et al.**	22	Case Report			0
**Guillen et al.**	23	Case Report			0
**Hasan Ahmad et al.**	24	Case Report			0
**Hsu et al.**	25	Case Report			0
**Huang et al.**	26	Case Report			0
**Jiang et al.**	27	Case Report			0
**Kates et al.**	28	Case Report			0
**Kemmner et al.**	29	Case Report			0
**Kim et al.**	30	Case Report			0
**Kocak et al.**	31	Case Report			0
**Kolonko et al.**	32	Case Report			0
**Kumar et al.**	33	Case Report			0
**Lauterio et al.**	34	Case Report			0
**Li.Q**	35	Case Report			0
**Ma et al.**	36	Case Report			0
**Machado et al.**	37	Case Report			0
**Man et al.**	38	Case Report			0
**Marx et al.**	39	Case Report			0
**Meziyerh et al.**	40	Case Report			0
**Namazee et al.**	41	Case Report			0
**Ning et al.**	42	Case Report			0
**Sakulkonkij et al.**	43	Case Report			0
**Seminari et al.**	44	Case Report			0
**Shingare et al.**	45	Case Report			0
**Sj Antony et al.**	46	Case Report			0
**Suwanwongse et al.**	47	Case Report			0
**Tanaka et al**	48	Case Report			0
**Tantisattamo et al.**	49	Case Report			0
**Thammathiwat et al.**	50	Case Report			0
**Tzukert T. et al.**	51	Case Report			0
**Velioglu et al.**	52	Case Report			0
**Wang et al.**	53	Case Report			0
**Wang et al.**	54	Case Report			0
**Xu et al.**	55	Case Report			0
**Zhong et al.**	56	Case Report			0

**Zhu et al.**	57	Case Report			0
**Zhu et al.**	58	Case Report			0
**Akalin et al.**	59	Case series			0
**Alberici et al.**	60	Case series			0
**Banerjee et al.**	61	Case series			0
**Bosch et al.**	62	Case series			0
**Chen et al.**	63	Case series			0
**Columbia University**	64	Case series			0
**Crespo et al.**	65	Case series			0
**Devresse et al.**	66	Case series			0
**Elias et al.**	67	Prospective study			0
**Fernandez-Riuz et al.**	68	Case series			0
**Fung et al.**	69	Case series			0
**Hartzell et al.**	70	Case series			0
**Lubetzky et al.**	71	Case series			0
**Maritati et al.**	72	Case series			0
**Mehta et al.**	73	Case series			0
**Mella et al.**	74	Case series			0
**Monfaret et al.**	75	Case series			0
**Nair et al**	76	Case series			0
**Pierotti et al.**	77	Case series			0
**Rodriguez-Cubillo et al.**	78	Case series			0
**Silva et al.**	79	Case series			0
**Trujillo et al.**	80	Case series			0
**Zhu et al.**	81	++++	+	++	7
